# 

**Published:** 1905-10-28

**Authors:** 


### NOTICE TO CORRESPONDENTS.

All MSS., Letters, Books for Review, and other matters intended for the Editor should be addressed The Editor, "The Hospital," The Hospital Buildings, 28 & 29 Southampton Street, Strand, W.C.

The Editor cannot undertake to return rejected MSS., even when accompanied by stamped directed envelope.

**Notice to Advertisers and Subscribers.**

All Advertisements, Orders for Copies of The Hospital, and Business Communications should be addressed to **THE MANAGER (Not to the Editor),** The Hospital, 28 & 29 Southampton Street, Strand, London, W.C.

The Cost of Subscription, post free, is at follows:—Per week, 3½d.; per quarter, 3s. 3d.; per half-year, 6s. 6d.; per annum, 13s. Foreign and Colonial:—Per annum, 19s. 6d., payable, in all cases, in advance.

**NOTICE.—Vol. XXXVII. of "The Hospital" October 1904 to March 1905), in handsome cloth binding, will shortly be on sale at the office of this Journal, price 10s. 6d. Cases for binding the Numbers contained in this Volume 2s. Index to Vol. XXXVII., 3½d., post free.**

**The Monthly Part for October is now ready. Price 1s., by post, 1s. 3d.**

### Medical Appointments.

**Bulkeley, L. A. H.**, M B., B.S.Dur., House Surgeon to the Royal Infirmary, Newcastle-upon-Tyne. **Dir gle, Percival Alfi ed**, L.R.C.P.Lond., M.R.C.S.Eng., House Physician to the Royal Hospital for Diseases of the Chest, City Road, E.C. **Edmiston, J. F.**, M.B., Ch B. Liverp., House Surgeon to the David Lewis Northern Hospital, Liverpool. **Gush, H. W.**, M.B., Ch.B.Edin., House Physician to the David Lewis Northern Hospital, Liverpool. **Hawksley, W. L.**, M.B.,Ch B.,Liverp., House Physician to the David Lewis Northern Hospital Liverpool. **Ia re ce, G.**, M.R.C.S., L.R.C.P.Lond., House Surgeon to the David Lewis Northern Hospital, Liverpool. **Lister, T. D.**, M.D., M.R.C.P.Lond., Physician to the Mount Vernon Hospital for Consumption and Diseases of the Chest, Hampstead and Northwood. **Lloyd, G. E.**, M.B., B.S.Dur., House Surgeon to the Royal Infirmary, Newcastle-upon-Tyne. **Lu n, W. E. C.**, Accident Room House Surgeon to the Royal Infirmary, Newcastle-upon-Tyne. **M'Be n, S. L.**, M.B., B.S.Durh,, House Surgeon to the Royal Infirmary, Newcastle-upon-Tyne. **McDowall**, M B., B.S.Durh., Junior House Physician to the Royal Infirmary, Newcastle-upon-Tyne. **Macrae, Ait k n D.**, M.A., M.B., Ch.B.Edin., F.R.C.S.Edin., Assistant Medical Officer at the Bolingbroke Hospital, S.W. **Mallam, H. G.**, M.R.C.S., L.R.C.P.Lond., Honorary Medical Officer to the Brighton, Hove, and Preston Dispensary. **Parsons, Walter Brock**, M.R.C.S., L.R.C.P.Lond., Second Honorary Anæsthetist to the North-West London Hospital. **Reah, H.**, M.B., B.S.Durh., House Surgeon to the Royal Infirmary, Newcastle-upon-Tyne. **Saunders, Ernest George Symes**, M.D., C.M.Aberd., Assistant Surgeon to the Royal Albert Hospital, Devonport. **Swan, R. H. J.**, M.B., M.S. Lond., F.R.C.S.Eng., Third Assistant Surgeon at the Cancer Hospital. **Yorke, C.**, M.B., Ch.B.Liverp., House Surgeon to the David Lewis Northern Hospital, Liverpool.

### SOME NEW BOOKS PUBLISHED BY THE SCIENTIFIC PRESS, LTD.

*NOW READY.*

### MEDICAL ELECTRICITY AND THE LIGHT TREATMENT.

**By KATE NEALE.**

Cloth, profusely Illustrated, 2/6 net, 2/9 post free.

A practical work on a subject hitherto untouched from a Nursing standpoint. The book cannot fail to be of the utmost value to all Nurses who desire to move with the times.

### THE NURSING OF SICK CHILDREN.

**By Dr. BURNET.**

Cloth, 1/- net, 1/1 post free.

A valuable treatise on the nursing of sick children. The book should prove useful to mothers as well as to nurses.

***Two Valuable Guides to those desirous of taking up Nursing as a Profession.***

### HOW TO BECOME A NURSE.

**THE NURSING PROFESSION:**
**How and Where to Train.**

Edited by Sir HENRY BURDETT, K.C.B.

Cloth, 2/- net, 2/4 post free.

An invaluable Guide to would-be Nurses. It gives full details of Training Schools, Premiums, Salaries, the Hours of Duty, &c. &c.

*JUST PUBLISHED.*

### THE DUTIES OF A NURSE.

**NURSING:**
**Hints to Probationers on Practical Work.**

By MARY H. ANNESLEY VOYSEY.

2/- net, 2/3 post free.

A practical guide to the duties of a nurse probationer. The work will prove of the greatest assistance, and should be read by all who are contemplating Nursing as a profession.

### HINTS TO NURSES ON TROPICAL FEVERS.

By S. F. POLLARD,
Sister Army Nursing Service Reserve, late Sister Guy's Hospital.

Cloth, 1/6 net, 1/8 post free.

Contents.

Hints on Going Abroad—Malaria—Dysentery—Liver Abscess—Cholera — Bubonic Plague — Beri-beri—Yellow Fever—Malta Fever—Various Fevers, &c.

### SIMPLE SANITATION:

**The Practical Application of the Laws of Health to Small Dwellings.**

By M. LOANE.

With Introductory Chapter on Sanitary Legislation by Dr. A. Mearns Fraser.

Cloth, Illustrated, 1/- net, 1/1 post free.

*READY SHORTLY.*

### SURGICAL INSTRUMENTS AND APPLIANCES USED IN OPERATIONS.

Classified and Illustrated Lists of Instruments in General Use and for Special Operations, with Explanatory Notes.

By HAROLD BURROWS, F.R.C.S.

Limp Cloth, 1/6 net, 1/8 post free.

A work of reference of the utmost value to ***all*** nurses.

THE BOOK FOR THE C.M.B.

### A COMPLETE HANDBOOK OF MIDWIFERY.

By J. K. WATSON, M.D. Edin.

143 Illustrations. 6/- net, 6/4 post free.

**The Scientific Press, Ltd.**

***Complete Catalogue sent post free on application.***

**Publishers of Nursing Text-Books, Charts, and Case-Books of all Descriptions.**

**28 & 29 SOUTHAMPTON STREET, STRAND, LONDON, W.C.**

